# Curiosity killed the cat: no evidence of an association between cat ownership and psychotic symptoms at ages 13 and 18 years in a UK general population cohort

**DOI:** 10.1017/S0033291717000125

**Published:** 2017-02-22

**Authors:** F. Solmi, J. F. Hayes, G. Lewis, J. B. Kirkbride

**Affiliations:** Division of Psychiatry, University College London, London, UK

**Keywords:** ALSPAC, cat ownership, pet ownership, psychosis, psychotic symptoms

## Abstract

**Background:**

Congenital or early life infection with *Toxoplasma gondii* has been implicated in schizophrenia aetiology. Childhood cat ownership has been hypothesized as an intermediary marker of *T. gondii* infection and, by proxy, as a risk factor for later psychosis. Evidence supporting this hypothesis is, however, limited.

**Method:**

We used birth cohort data from the Avon Longitudinal Study of Parents and Children (ALSPAC) to investigate whether cat ownership in pregnancy and childhood (ages 4 and 10 years) was associated with psychotic experiences (PEs) in early (age 13, *N* = 6705) and late (age 18, *N* = 4676) adolescence, rated from semi-structured interviews. We used logistic regression to examine associations between cat ownership and PEs, adjusting for several sociodemographic and socioeconomic factors, household characteristics and dog ownership. Missing data were handled via multiple imputation.

**Results:**

Cat ownership during pregnancy was not associated with PEs at age 13 years [adjusted odds ratio (OR) 1.15, 95% confidence interval (CI) 0.97–1.35] or 18 years (OR 1.08, 95% CI 0.86–1.35). Initial univariable evidence that cat ownership at ages 4 and 10 years was associated with PEs at age 13 years did not persist after multivariable adjustment (4 years: OR 1.18, 95% CI 0.94–1.48; 10 years: OR 1.12, 95% CI 0.92–1.36). There was no evidence that childhood cat ownership was associated with PEs at age 18 years.

**Conclusions:**

While pregnant women should continue to avoid handling soiled cat litter, given possible *T. gondii* exposure, our study strongly indicates that cat ownership in pregnancy or early childhood does not confer an increased risk of later adolescent PEs.

## Introduction

House cats are the primary hosts of *Toxoplasma gondii*, a protozoan parasite that can infect various warm-blooded animals, including humans (Tenter *et al.* 2000; Webster *et al.* 2013). Infection can occur *in utero* or postnatally, via ingestion of either the parasite's oocysts – which might be present in soil, water, or food – or tissue cysts from infected animals (e.g. in raw or undercooked meat). In intermediate hosts (e.g. humans or animals other than cats), the parasite exploits lymphocytes to encroach in muscle tissues and, importantly, the brain, where it can form tissue cysts in neurons, microglia, and astrocytes (Carruthers & Suzuki, 2007).

Although the evidence is not unequivocal (Sugden *et al.* 2016), data from several epidemiological, experimental, and animal studies suggests that *T. gondii* infection may be implicated in the aetiology of psychosis. For example, dopaminergic dysfunction and cognitive impairments – similar to those observed in people with schizophrenia – have been observed in infected rodents (Gaskell *et al.* 2009; Prandovszky *et al.* 2011; McConkey *et al.* 2013) and humans (Kannan & Pletnikov, 2012); these people may also experience hallucinations during acute infection with the parasite (Sugden *et al.* 2016). A recent meta-analysis of 38 studies found that compared with controls, people with schizophrenia were nearly three times more likely to be seropositive for *T. gondii* antibodies [odds ratio (OR) 2.71, 95% confidence interval (CI) 1.93–3.80] (Torrey *et al.* 2012). Higher seroprevalence and serointensity of *T. gondii* IgG (but not IgM, an indicator of recent infection) in people with schizophrenia (Cetinkaya *et al.* 2007) and their mothers (Brown *et al.* 2005; Mortensen *et al.* 2007*a*) suggest that either early life exposure to the parasite, congenital infection, or transmission of maternal antibodies could alter neurodevelopment of subsequent offspring.

Assuming a causal relationship between *T. gondii* infection and later psychosis, some researchers have hypothesized that cat ownership should confer an increased risk of psychotic disorders (Torrey & Yolken, 1995; Yuksel *et al.* 2010; Torrey *et al.* 2015). Moreover, this theory has been proposed to explain several epidemiological findings, including higher rates of psychotic disorders in urban populations (with higher cat densities and subsequent possibility for infection) (Torrey & Yolken, 1995; Torrey *et al.* 2000, 2015). Nonetheless, robust empirical evidence supporting this theory remains limited. Cat ownership or contact during pregnancy (Kapperud *et al.* 1996; Cook *et al.* 2000) and childhood (Taylor *et al.* 1997) do not appear to be associated with *T. gondii* infection, although handling soiled cat litter is known to be associated with infection (Kapperud *et al.* 1996). Epidemiological studies which have reported an association between cat ownership and psychosis (Torrey & Yolken, 1995; Torrey *et al.* 2000, 2015; Yuksel *et al.* 2010), have generally been hindered by notable methodological limitations (Wolf & Hamilton, 2015), including reliance on case-control designs that are susceptible to recall bias, small *ad hoc* samples and weak statistical analyses, which have failed to adequately account for confounding or missing data.

To overcome these issues, we sought to test whether prenatal and childhood cat ownership were associated with an increased risk of developing psychotic experiences (PEs) in early and late adolescence, using longitudinal data from the Avon Longitudinal Study of Parents and Children (ALSPAC). PEs in adolescence are an established risk factor for later schizophrenia, particularly with respect to psychotic symptoms which emerge or persist in late adolescence (Poulton *et al.* 2000; Fisher *et al.* 2013). Since *T. gondii* infection is proposed to increase psychosis risk by affecting early life neurodevelopment (Mortensen *et al.* 2007*b*), we restricted cat ownership to the prenatal and early childhood period (at age 4 years), although we also performed additional analyses using cat ownership at age 10 years to better align with previous research (Torrey & Yolken, 1995; Torrey *et al.* 2000, 2015; Yuksel *et al.* 2010).

## Method

### Sample

The ALSPAC study invited 16 734 pregnant women expected to deliver between 1 April 1991 and 31 April 1992 resident in the former county of Avon in the Southwest of England to participate; of these, 14 541 (87%) enrolled resulting in 14 062 live births and 13 988 children alive at age 1 year. The sample was supplemented with 713 additional children (whose mothers were originally eligible for the study) during follow-up between the ages of 7 and 18 years. More details on recruitment, follow-up assessments and time-points have been published elsewhere (Boyd *et al.* 2012). All mothers gave informed written consent prior to recruitment and the ALSPAC Ethics and Law Committee and the Local Research Ethics Committees gave ethical approval for this study.

In this study we included children with complete data on PEs at ages 13 (*N* = 6705) and 18 (*N* = 4676) years. For twin pairs (*N* = 87, 2.6% of the sample at age 12, and *N* = 42, 1.8% at age 18), we excluded one sibling (sibling ‘B’) to avoid biasing estimates due to shared genetic and environmental exposures; there is no evidence that, in twins, birth order is related to schizophrenia risk (Onstad *et al.* 1992; Kleinhaus *et al.* 2008).

### Exposure variable

Information on pet ownership was reported by mothers via postal questionnaires during pregnancy, and subsequently when their child was aged 8, 21, 31 and 47 months. In addition to cat ownership, mothers were asked about the number and type of other pets owned, including: dogs, rabbits, rodents, birds (all waves), and tortoises and fish (from 21 months). From these questions we created two primary binary exposure variables, indicating whether the mother owned a cat (yes/no): (i) in pregnancy; and (ii) at child's age of 47 months (~4 years). As a secondary exposure, we employed cat ownership at 10 years (122 months) in order to create an exposure variable comparable with those used in previous studies (Torrey & Yolken, 1995; Torrey *et al.* 2000, 2015). We did not test whether a dose-response effect existed between duration of cat ownership and psychotic symptoms, since the majority (89%) of children who reported cat ownership at age 4 years also owned one at ages 8, 21, and 31 months, as reported by their mothers.

### Outcome variables

At approximately ages 13 and 18 years, children attended clinic visits where they were administered the psychotic-like symptoms interview (PLIKSi), a semi-structured interviewer-rated screening assessment for PEs. The PLIKSi contains six questions on unusual experiences (i.e. derealization, depersonalization, self-unfamiliarity, dysmorphophobia, partial object perception, and other perceptual abnormalities) followed by 12 questions adapted from the Diagnostic Interview Schedule for Children version IV (DISC-IV; Shaffer *et al.* 2000) and the Schedule for Clinical Assessment in Neuropsychiatry (SCAN; WHO, 1994). These are aimed at assessing the presence of delusions (being spied on, persecuted, having thoughts read, reference, control, grandiose ability, and other delusions), hallucinations (visual and auditory), and intrusive thoughts (thought broadcasting, insertion and withdrawal) (Horwood *et al.* 2008). Total scores were recoded into a binary variable indicating the absence, or the suspected/definite presence of symptoms, consistent with previous investigations of PEs in ALSPAC (Horwood *et al.* 2008; Dorrington *et al.* 2014). Children whose psychotic symptoms could have been attributed to fever or sleep problems were coded as not having the outcome (Zammit *et al.* 2008, 2009; Dorrington *et al.* 2014).

### Other variables

We employed causal diagrams, known as directed acyclic graphs (DAGs; Textor & Liśkiewicz, 2011), to identify variables which could confound the association between cat ownership and psychotic symptoms. We modelled hypothesized associations between a broad initial set of potential child- and mother-based variables, cat ownership in either pregnancy or childhood and PEs using the DAGitty web-based software (Textor *et al.* 2011). Our DAGs (Supplementary Figs S1 and S2) suggested that it was inappropriate to control for some of these variables, either because they did not meet criteria for confounding (e.g. child gender, stressful life events, maternal depression, pet ownership other than dogs), or because adjustment for other variables (e.g. paternal age, dog ownership in pregnancy) provided sufficient control for any other causal paths (e.g. maternal age, dog ownership at age 4 years). From our DAGs we were able to identify the minimal sufficient number of confounders of the relationship between exposure to cat ownership in pregnancy and childhood and PEs at ages 13 and 18 years. These included: child ethnicity (white/non-white – including Black African, Black Caribbean, Other Black, Indian, Pakistani, Bangladeshi, Chinese, Other, mixed); paternal age (at the time of mother's pregnancy); maternal marital status in pregnancy (single, separated, divorced, or widowed/married); highest maternal academic education in pregnancy (vocational course/secondary schooling/university degree or higher); maternal social class (manual *v*. non-manual profession); number of house moves up to age 47 months (~4 years); housing type (detached, semi-detached, terraced/flat, other); household crowding index (range 0–1); and dog ownership in pregnancy.

The ALSPAC website contains details of all the data that is available through a fully searchable data dictionary (available at: http://www.bris.ac.uk/alspac/researchers/data-access/data-dictionary/).

### Statistical analyses

We employed cross-tabulations (with χ² tests) and ANOVAs to (i) investigate the presence of selective attrition in the sample, by comparing children with and without missing outcome data on exposure and confounding variables; (ii) describe the sample and test for differences in the distribution of exposure and confounding variables across outcome levels. We then fitted univariable and multivariable logistic regression models adjusting for all variables identified as relevant confounders using DAGs (Supplementary Figs S1 and S2), i.e.: dog ownership in pregnancy; housing type; household crowding; maternal education, social class, and marital status; paternal age; number of house moves. When the studied exposure was cat ownership in childhood, we further adjusted for maternal cat ownership in pregnancy.

Since our primary hypothesis was a null association between cat ownership and psychotic symptoms, we conducted power calculations, assuming an alpha of 0.05 to determine the effect size we could reasonably expect to detect, had it existed, given our sample sizes. Our sample had over 90% power to detect an OR of 1.25 based on the observed exposure distribution and sample sizes.

### Missing data

Analyses were based on participants with complete data at age 13 (*N* = 6705) and 18 (*N* = 4676) years. Missing main exposures and covariate data varied between 0.04% (pregnancy) to 34.08% (age 4 years), and 0.02% (pregnancy) to 33.58% (age 4 years) at each time point, respectively (Supplementary Table S1).

We used multiple imputation with chained equations (MICE) and the Stata *ice* command (Royston & White, 2011) to impute missing exposure and covariate data, including all observed exposure, covariate and outcome data into our multiple imputation (MI) routine, in addition to several auxiliary variables which could provide information about missing values. These included: gender; stressful life events; maternal depression in pregnancy; other pet (rabbit, rodents, turtles, birds, fish) ownership in pregnancy and age 4 years; two measures of depressive symptoms at ages 12 and 18 years assessed via self-reported with the short moods and feelings questionnaire (SMFQ; Angold *et al.* 1995); a continuous measure of IQ assessed during a clinical assessment at age 8 years using the Wechsler Intelligence Scale for Children – third edition (WISC-3); and a measure of family income at child's age of 33 months. We also included a variable indicating maternal history of schizophrenia at birth in light of the known genetic heritability of psychosis (Lichtenstein *et al.* 2009). We imputed 100 datasets using linear, logistic, ordinal logistic, and multinomial logistic models according to the nature of the variables whose missing values had to be imputed. As sensitivity analyses we examined the association between cat ownership and adolescent PEs in (i) a complete case analysis, to assess any possible bias introduced when failing to account for missing data, and (ii) using data on the full ALSPAC sample (*N* = 15 023) with multiple imputation on those missing outcome data, to assess possible biases introduced by our main choice of multiple imputation.

All analyses were performed using Stata v. 13 (StataCorp, 2013).

## Results

### Missing data

Children with missing outcome data at ages 13 and 18 years were more likely to be boys, from a non-white ethnic background, to live in more crowded houses, and have experienced a stressful life event by age 4 years. Children with missing data were also more likely to have younger parents, and a mother who had suffered from probable depression in pregnancy, was less well educated, not married, from a manual occupation, and who had moved house more frequently. At ages 13 and 18 years, children whose mother had owned a cat in pregnancy were less likely to have missing data, although children whose mother had owned a cat in their childhood were more likely to have missing outcome data at age 13 years. Participants with missing outcome data at either age were more likely to have a mother who owned a dog, a bird, a rabbit, or rodent in pregnancy or during childhood (Supplementary Table S1).

### Sample characteristics

A total of 6705 and 4676 children had complete data on psychotic symptoms at ages 13 and 18 years, respectively, and were therefore included in the analyses. At both ages, the majority of each sample was of female gender, white ethnicity, had a mother who had completed at least A-levels, was married, non-manual profession, and had moved house <3 times during the 4 years prior to pregnancy and age 4 years of the child (Table 1) In both samples, around one third of mothers owned a cat during pregnancy, and at 4 and 10 years (Table 1). Among children who owned a cat at age 4 years, between 79% and 89% also owned a cat at previous waves of data collection (i.e. at ages 8, 21, and 33 months). Among those who owned a cat at age 10 years, between 62% and 86% also owned a cat at a previous time point (data available from authors).

**Table 1.** Sample characteristics

Variables	Psychotic experiences age 13 years				Psychotic experiences age 18years			
	*N* (%) *N* = 6705	None, *N* (%) *N* = 5929 (88.43%)	Suspected or definite, *N* (%), *N* = 776 (11.57%)	*p* (χ^2^)	*N* (%) (*N* = 4676)	None, *N* (%) *N* = 4306 (92.09%)	Suspected or definite, *N* (%), *N* = 370 (7.91%)	*p* (χ^2^)
Cat ownership in pregnancy (yes)	1959 (31.66)	1719 (31.35)	240 (34.09)	0.14	1336 (31.17)	1227 (31.06)	109 (32.44)	0.60
Cat ownership at age 4 (yes)	1620 (28.78)	1407 (28.21)	213 (33.23)	0.008	1017 (28.54)	1019 (28.47)	88 (29.33)	0.75
Cat ownership at age 10 (yes)	1837 (31.83)	1611 (31.40)	226 (35.26)	0.05	1232 (31.44)	1134 (31.22)	98 (34.15)	0.30
Dog ownership in pregnancy (yes)	1380 (22.30)	1209 (22.05)	171 (24.29)	0.18	880 (20.53)	800 (20.25)	80 (23.81)	0.79
Gender (female)	3420 (51.03)	2995 (50.54)	425 (54.77)	0.03	2639 (56.45)	2401 (55.77)	238 (64.32)	0.001
Ethnicity (Non-white)	239 (3.95)	209 (3.90)	30 (4.34)	0.58	180 (4.30)	159 (4.11)	21 (6.56)	0.04
Social class (manual)	833 (15.62)	728 (15.39)	105 (17.38)	0.20	568 (15.22)	507 (14.69)	61 (21.71)	0.002
Housing type (flat)	812 (13.19)	685 (12.55)	127 (18.14)	<0.0001	562 (13.16)	495 (12.57)	67 (20.12)	<0.0001
Marital status (married)	5046 (81.24)	4523 (82.13)	523 (74.29)	<0.0001	3516 (81.63)	3286 (82.73)	230 (68.66)	<0.0001
Maternal education								
Up to A level	3823 (62.28)	3366 (61.97)	457 (64.73)	0.03	2628 (61.79)	2437 (62.17)	191 (57.36)	<0.0001
Degree or higher	1005 (16.37)	914 (16.83)	91 (12.89)		822 (19.33)	771 (19.67)	51 (15.32)	
Number of house moves								
1–3	1417 (29.23)	1245 (29.00)	172 (31.39)	0.05	993 (29.24)	912 (29.02)	81 (32.02)	0.09
* ⩾*4	629 (13.00)	545 (12.67)	84 (15.33)		428 (12.60)	388 (12.34)	40 (15.81)	
Crowding index								
0.25%	1892 (31.03)	1685 (31.16)	207 (29.96)	0.02	1278 (30.18)	1179 (30.20)	99 (30.00)	<0.0001
0.75%	929 (15.23)	814 (15.05)	115 (16.64)		599 (14.15)	532 (13.63)	67 (20.30)	
1%	241 (3.95)	200 (3.70)	41 (5.93)		161 (3.80)	138 (3.53)	23 (6.97)	
		Mean s.d.	Mean s.d.		Mean s.d.	Mean s.d.	Mean s.d.	
Maternal age, years	29.18 (4.52)	28.79 (4.51)	28.19 (4.72)	0.001	29.42 (4.57)	29.03 (4.53)	27.69 (5.23)	<0.0001
Paternal age, years	30.71 (6.72)	31.33 (5.46)	31.14 (5.53)	0.46	31.10 (6.76)	31.59 (5.52)	30.54 (5.40)	0.005

At age 13 years, a greater proportion of the sample who reported suspected/definite psychotic symptoms owned a cat in childhood, were girls, lived in flats and in crowded home environments, had moved homes more frequently, and had a mother who was younger, less well educated, and of single marital status (Table 1). Similar patterns were generally observed for the sample at age 18 years (Table 1), although there was no longer any apparent association between PEs and number of house moves, while non-manual social class and non-white ethnicity were associated with experiencing suspected/definite PEs at this age.

### Cat ownership and psychotic symptoms

Cat ownership in pregnancy was not associated with psychotic symptoms at age 13 or 18 years in either univariable (age 13: OR 1.15, 95% CI 0.97–1.35; age 18; OR 1.08, 95% CI 0.86–1.35) or in multivariable (age 13: adjusted OR 1.15, 95% CI 0.97–1.36; age 18; OR 1.08, 95% CI 0.85–1.37) models, following multiple imputation (Table 2). Owning a cat at age 4 years was associated with higher odds of having PEs at age 13 years in univariable models (OR 1.23, 95% CI 1.04–1.46), but this effect was no longer significant after multivariable adjustment (OR 1.18, 95% CI 0.94–1.48). There was no evidence that cat ownership at age 4 years was associated with PEs at age 18 years (univariable OR 1.11, 95% CI 0.88–1.40; adjusted OR 0.97, 95% CI 0.71–1.31). These patterns were similar with respect to cat ownership at age 10 years, with no apparent association with PEs at age 13 years (OR 1.12, 95% CI 0.92–1.36) or 18 years (OR 1.08, 95% CI 0.82–1.45) after multivariable adjustment (Table 2).

**Table 2.** Univariable and multivariable odds ratios (OR) and 95% confidence intervals (CI) for the association between maternal cat ownership in pregnancy and between the ages of 8 months and 4 years of the child and psychotic symptoms (suspected or definite v. none) at ages 13 and 18 years (N includes exposure and confounding variables imputed with multiple imputation with chained equations, N = 100 imputations)

	Psychotic experiences age 13 (suspected or definite *v*. none) *N* = 6705	
	Crude OR (95% CI)	Adjusted OR^a^ (95% CI)
**Exposure variable**		
Cat ownership in pregnancy		
No	Ref.	Ref.
Yes	1.15 (0.97–1.35)	1.15 (0.97–1.36)
Cat ownership at age 4		
No	Ref.	Ref.
Yes	1.23 (1.04–1.46)**	1.18 (0.94–1.48)
Cat ownership at age 10		
No	Ref.	Ref.
Yes	1.19 (1.00–1.41)**	1.12 (0.92–1.36)

	Psychotic experiences age 18 (suspected or definite *v*. none) *N* = 4676	
	Crude OR (95% CI)	Adjusted OR^a^ (95% CI)
Cat ownership in pregnancy		
No	Ref.	Ref.
Yes	1.08 (0.86–1.35)	1.08 (0.85–1.37)
Cat ownership at age 4		
No	Ref.	Ref.
Yes	1.11 (0.88–1.40)	0.97 (0.71–1.31)
Cat ownership at age 10		
No	Ref.	Ref.
Yes	1.15 (0.89–1.48)	1.08 (0.82–1.45)

** *p* < 0.05.

a Model of cat ownership in pregnancy is adjusted for child ethnicity; maternal education, marital status, and social class; paternal age; number of house moves until age 4, type of house, crowding index. Models of cat ownership in at ages 4 and 10 years are further adjusted for cat ownership in pregnancy.

### Sensitivity analyses

In complete case analyses cat ownership in pregnancy was associated with higher odds of PEs at age 13 years in all models (univariable OR 1.31, 95% CI 1.04–1.65; adjusted OR 1.34, 95% CI 1.06–1.69), as was cat ownership at age 4 years (univariable OR 1.44, 95% CI 1.13–1.83; adjusted OR 1.47, 95% CI 1.01–2.13). Cat ownership at age 10 years was only associated with PEs at age 13 years in univariable models (OR 1.30, 95% CI 1.02–1.66), but not in adjusted models. We found no evidence of an association between cat ownership and PEs at age 18 years (Supplementary Table S3).

Next, we used the fully imputed dataset to examine the association between cat ownership and adolescent PEs on the whole ALSPAC cohort (*N* = 15 023). The pattern of these results (Supplementary Table S4) was very similar to the magnitude, direction and general lack of association between cat ownership and adolescent PEs reported in our main results based on imputation of exposures and confounders (Table 2). Together these sensitivity analyses suggested that complete case analyses may lead to biased risk estimates, while our choice of MI routine did not substantially bias our results.

## Discussion

We found no evidence that cat ownership in pregnancy or childhood was associated with PEs in early and late adolescence using prospectively collected data from a large population-based cohort, following control for several confounders and methods that investigate the likely impact of missing data.

Our findings in relation to PEs are not consistent with the existing literature that has studied cat ownership in people with schizophrenia (Torrey & Yolken, 1995; Torrey *et al.* 2000, 2015; Yuksel *et al.* 2010). We suggest that several methodological differences between our study and other investigations, including previous reliance on small, retrospective, convenience samples, may explain the discrepancy.

Our study was based on PEs in early and late adolescence, unlike other studies which were based on a clinical diagnosis of schizophrenia (Torrey & Yolken, 1995; Torrey *et al.* 2000, 2015; Yuksel *et al.* 2010). One possible explanation of our null findings is that cat ownership does not affect the population expression of psychosis (i.e. does not shift the continuum), but operates only to increase risk of threshold symptoms for clinical disorder. We consider this explanation unlikely, given that: psychotic symptoms in late childhood and adolescence predict onset of non-affective psychosis and other psychopathology (Poulton *et al.* 2000; Laurens *et al.* 2007; Kelleher *et al.* 2012; Fisher *et al.* 2013) and psychotic symptoms in late adolescence are better predictors of future psychopathology. In line with other literature (Kelleher *et al.* 2012), we observed a decline in the prevalence of PEs between ages 13 and 18 years. If symptoms in later adolescence therefore provide a greater indication of later clinical disorder (Poulton *et al.* 2000; Fisher *et al.* 2013) then our results at age 18 (where there was no evidence of any association between cat ownership and PEs) may have the strongest implications for likely effects of early life cat ownership on clinical disorder.

Unlike previous studies which investigated cat ownership up until ages 10 or 13 years (Torrey & Yolken, 1995; Torrey *et al.* 2000, 2015; Yuksel *et al.* 2010), we restricted our main exposure variables to cat ownership during potentially sensitive windows of neurodevelopment, namely pregnancy and at age 4 years (47 months). We included cat ownership at age 10 years as a secondary exposure, consistent with previous literature, but our results mirrored those found at age 4 years, generally indicating an absence of association. Our study was sufficiently powered to detect effect sizes previously observed in the schizophrenia literature with respect to cat ownership (ORs ⩾1.25) (Torrey & Yolken, 1995; Torrey *et al.* 2000, 2015; Yuksel *et al.* 2010), as evidenced by the initial univariable associations we only observed between childhood exposure and PEs at age 13 years. While our study would have had less power to detect smaller ORs, including those we observed between 1.04 and 1.15, any such small effects, if true, would not warrant particular public mental health attention.

Previous reports of positive associations between cat ownership and schizophrenia may therefore have been attributable to Type I error, particularly given the small sample sizes and lack of control for confounders inherent to some studies. We adjusted for several, theoretically-informed confounders, including ethnicity (Westgarth *et al.* 2010; Kirkbride *et al.* 2012), maternal academic achievement and social class (Mulvany *et al.* 2001; Werner *et al.* 2007; Westgarth *et al.* 2010), and parental age (Sipos *et al.* 2004; Lopez-Castroman *et al.* 2010; Westgarth *et al.* 2010; Petersen *et al.* 2011). We also adjusted for number of house moves in light of evidence of an association between residential mobility and PEs (Singh *et al.* 2014), crowding index and housing type as a proxies for both social class and greater possibility of contact with *T. gondii*-contaminated litter, and dog ownership as a possible confounder of the association between *T. gondii* infection (given an increased likelihood to contaminated soils outdoors) and psychosis risk.

Earlier studies also relied on retrospective recall, and hence the potential of recall bias, of cat exposure and did not distinguish between ownership in infancy *v*. later childhood, making it impossible to attribute risk to specific periods of cat ownership over the early life-course.

Finally, we employed multiple imputation techniques in order to account for missing data, which could have otherwise biased our results. Consistent with guidelines (Sterne *et al.* 2009), we included all known exposure, outcome, covariate and auxiliary variables in MI as well as additional variables which had been previously found to be associated with PEs in this sample. Comparing our results with those from complete case analyses suggests that selective participation may potentially bias estimates, and could therefore explain previous positive findings in the literature (Torrey & Yolken, 1995; Torrey *et al.* 2000, 2015). This hypothesis is further supported by our results using the fully-imputed sample, where no significant associations were found in line with our main findings.

In conclusion, there is good evidence to support an association between *T. gondii* infection and later risk of experiencing psychosis, and this research is consistent with possible inflammatory causes of schizophrenia and other psychotic disorders. From a public health perspective, however, it is perhaps reassuringly that data from our prospective longitudinal study were not consistent with the hypothesis that cat ownership in pregnancy or early childhood is a risk factor for later psychosis.

## Supplementary material

For supplementary material accompanying this paper visit http://dx.doi.org/10.1017/S0033291717000125. click here to view supplementary material
