# Supplementary material for: Curiosity killed the cat: no evidence of an association between cat ownership and psychotic symptoms at ages 13 and 18 years in a UK general population cohort
Source: Psychol Med. 2017 Feb 22;47(9):1659–67. doi: 10.1017/S0033291717000125 (PMC5939988; doi:10.1017/S0033291717000125)
Supplement: Supplementary file 1 [file S0033291717000125sup001.docx]

**Figure S1: Directed Acyclic Graph (DAG) used to identify confounders of the association between cat ownership in pregnancy and psychotic experiences at age 13 or 18 years**


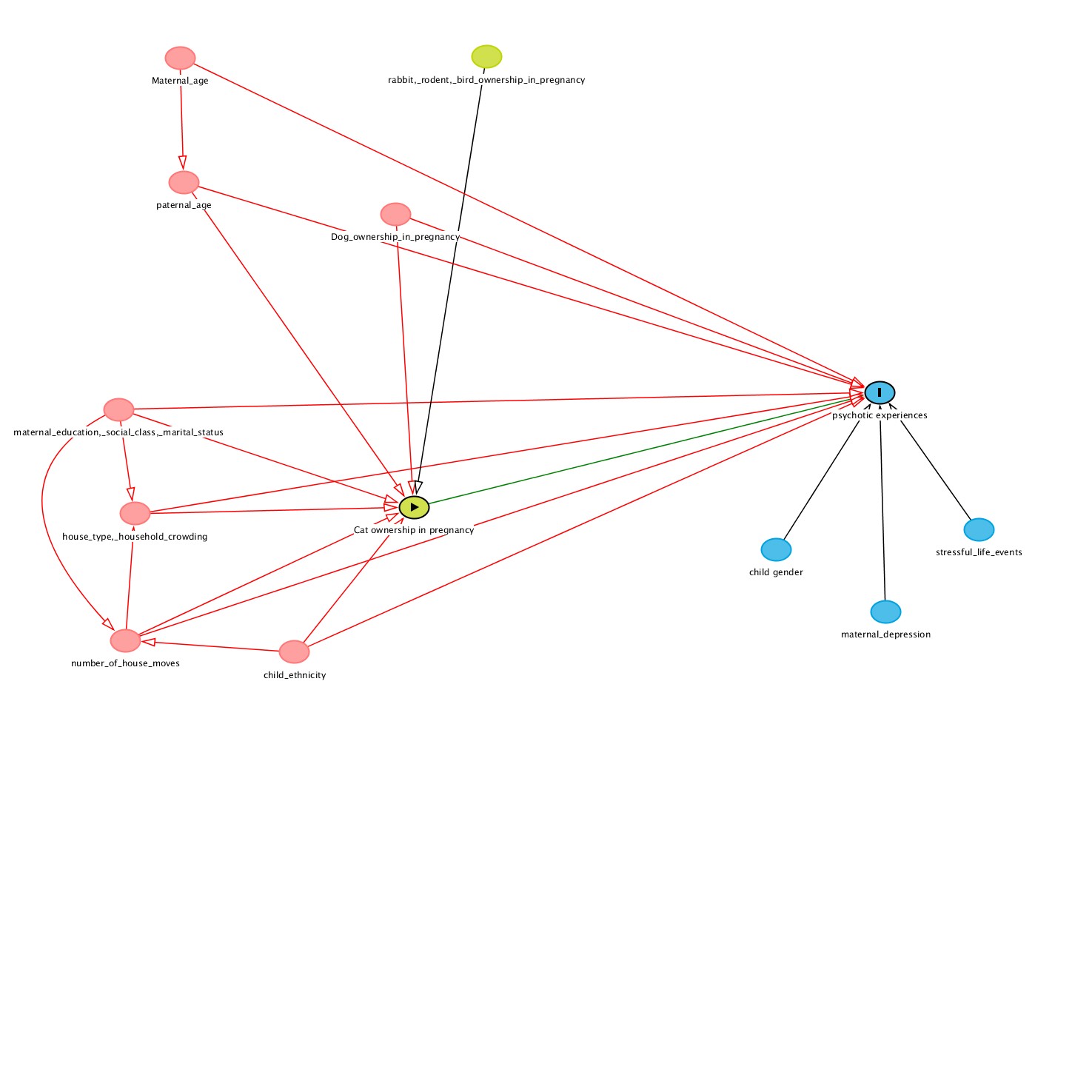


Figure Legend: Directed Acyclic Graphs were constructed using [www.dagitty.net](http://www.dagitty.net), and allow for identification of a minimum set of variables required to control for putative confounding in a theoretical model. Here, the green circle (with triangle) is the main exposure (cat ownership in pregnancy), with potential confounders in red circles (including, for example, dog ownership in pregnancy, which might have indicated a greater proportion of time outdoors and thus exposed to soil contaminated with *T.gondii*). The second green circle shows other variables hypothesised to be associated with the exposure but not the outcome (i.e. other types of pet ownership). Our outcomes (psychotic experiences) are denoted by the blue circle with the “I”. Other blue circles denote variables associated with psychotic experiences, but which would not be expected to be associated with our exposure (i.e. gender, maternal depression, stressful life events). Arrows denote the direction of causal association. From this DAG, we can identify the minimal set of confounders (to control for all confounding paths) to estimate the total effect of cat ownership in pregnancy on psychotic experiences as: Dog ownership in pregnancy; housing type; household crowding; maternal education, social class, and marital status; paternal age; number of house moves.

**Figure S2: Directed Acyclic Graph (DAG) used to identify confounders of the association between cat ownership at age four years and psychotic experiences at age 13 or 18 years.**


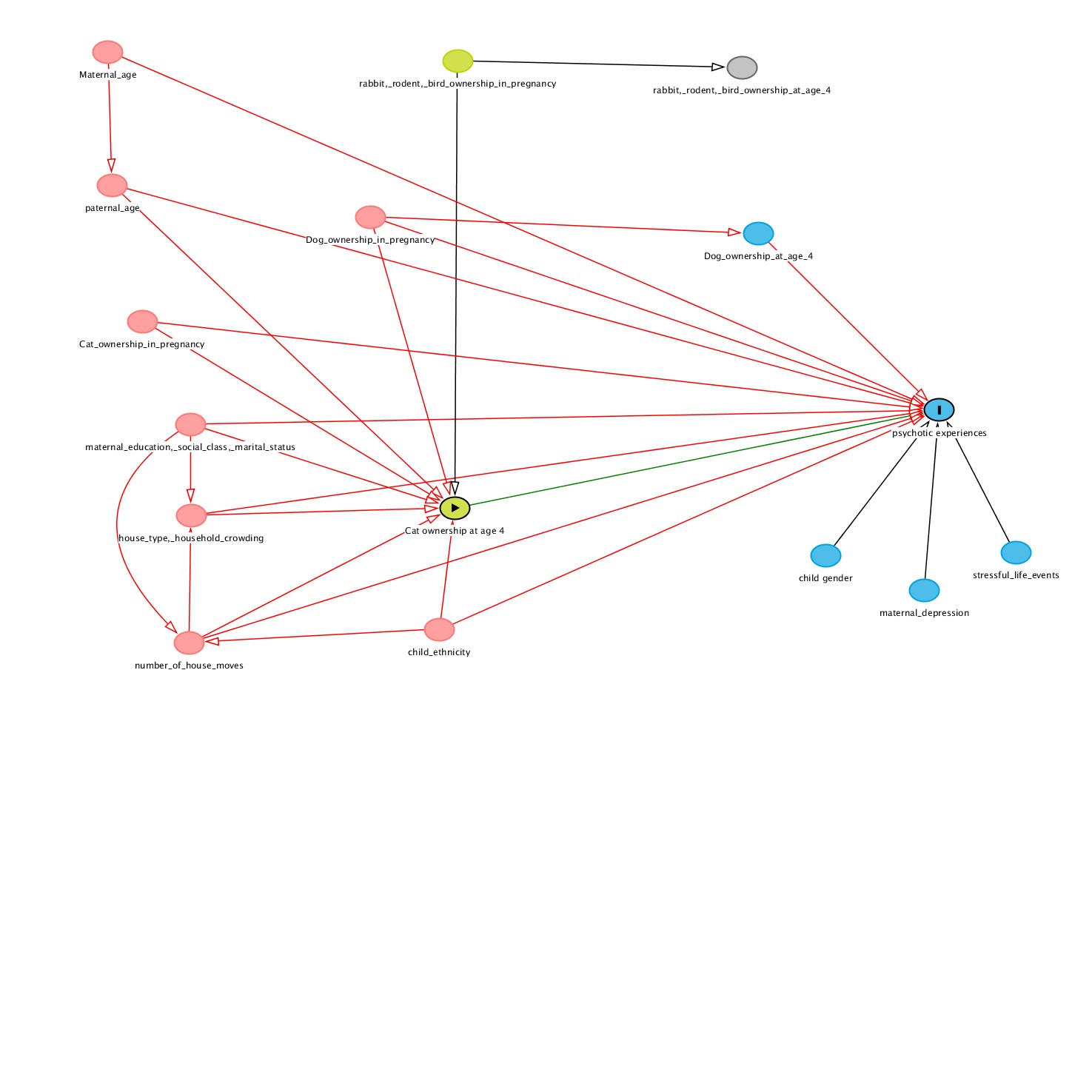


Figure Legend: Directed Acyclic Graphs were constructed using [www.dagitty.net](http://www.dagitty.net), and allow for identification of a minimum set of variables required to control for putative confounding in a theoretical model. Here, the green circle (with triangle) is the main exposure (cat ownership at age 4), with potential confounders in red circles (including, for example, dog ownership at pregnancy, which might have indicated a greater proportion of time outdoors and thus exposed to soil contaminated with *T.gondii*). Here, dog ownership in pregnancy may have either a direct or indirect effect on psychosis risk (via continued dog ownership at age 4). The second green circle shows other variables hypothesised to be associated with the exposure but not the outcome (i.e. other types of pet ownership at pregnancy). Our outcomes (psychotic experiences) are denoted by the blue circle with the “I”. Other blue circles denote variables associated with psychotic experiences, but which would not be expected to be associated with our exposure (i.e. gender, maternal depression, stressful life events). Arrows denote the direction of causal association. From this DAG, we can identify the minimal set of confounders (to control for all confounding paths) to estimate the total effect of cat ownership at age 4 on psychotic experiences as: Dog ownership in pregnancy; cat ownership in pregnancy; housing type; household crowding; maternal education, social class, and marital status; paternal age; number of house moves.

Table S1: Distribution (frequency and proportions) of exposure and other variables used in multiple imputation by outcome missingness at age 13 and 18 years.

| **Variables** | **Psychotic experiences age 13** | | | **Psychotic experiences age 18** | | |
| --- | --- | --- | --- | --- | --- | --- |
|  | **Missing**  **N (%)** | **Complete,**  **N (%)** | **P (χ²)** | **Missing**  **N (%)** | **Complete,**  **N (%)** | **P (χ²)** |
| ***Total*** | 8,538 (56.01%) | 6,705 (43.99%) |  | 10,567 (69.32%) | 4,676 (30.68%) |  |
| ***Cat Ownership in pregnancy (N=13,441)*** |  |  |  |  |  |  |
| *No* | 5,234 (72.15%) | 4,228 (68.34%) | <0.0001 | 6,512 (71.13%) | 2,950 (68.83%) | 0.006 |
| *Yes* | 2,020 (27.85%) | 1,959 (31.66%) |  | 2,643 (28.87%) | 1,336 (31.17%) |  |
| ***Cat Ownership at age 4 (N=9,448)*** |  |  |  |  |  |  |
| *No* | 2,714 (71.07%) | 4,009 (71.22%) | 0.87 | 3,951 (70.95%) | 2,772 (71.46%) | 0.58 |
| *Yes* | 1,105 (28.93%) | 1,620 (28.78%) |  | 1,618 (29.05%) | 1,107 (28.54%) |  |
| ***Cat Ownership at age 10 (N=8,035)*** |  |  |  |  |  |  |
| *No* | 1,554 (68.64%) | 3,934 (68.17%) | 0.68 | 2,801 (68.05%) | 2,687 (68.56%) | 0.62 |
| *Yes* | 710 (31.36%) | 1,837 (31.83%) |  | 1,315 (31.95%) | 1,232 (31.44%) |  |
| ***Dog ownership in pregnancy (N=13,441)*** |  |  |  |  |  |  |
| *No* | 5,286 (72.87%) | 4,807 (77.70%) | <0.0001 | 6,687 (73.04%) | 3,406 (79.47%) | <0.0001 |
| *Yes* | 1,968 (27.13%) | 1,380 (22.30%) |  | 2,468 (26.96%) | 880 (20.53%) |  |
| ***Rodent ownership in pregnancy (N=13,441)*** |  |  |  |  |  |  |
| *No* | 6,785 (95.53%) | 5,863 (94.33%) | <0.0001 | 8,564 (93.54%) | 4,057 (94.66%) | 0.012 |
| *Yes* | 469 (6.47%) | 351 (5.67%) |  | 591 (6.46%) | 229 (5.34%) |  |
| ***Bird ownership in pregnancy (N=13,441)*** |  |  |  |  |  |  |
| *No* | 6,654 (91.73%) | 5,742 (92.81%) | 0.020 | 8,398 (91.73%) | 3,998 (93.28%) | 0.002 |
| *Yes* | 600 (8.27%) | 445 (7.19%) |  | 757 (8.275) | 288 (6.72%) |  |
| ***Rabbit ownership in pregnancy (N=13,441)*** |  |  |  |  |  |  |
| *No* | 6,589 (90.83%) | 5,682 (91.84%) | 0.039 | 8,316 (90.84%) | 3,955 (92.28%) | 0.006 |
| *Yes* | 665 (9.17%) | 505 (8.16%) |  | 839 (9.16%) | 331 (7.72%) |  |
| ***Dog ownership at age 4 (N=9,448)*** |  |  |  |  |  |  |
| *No* | 2,927 (76.64%) | 4,587 (81.49%) | <0.0001 | 4,289 (77.02%) | 3,225 (83.14%) | <0.0001 |
| *Yes* | 892 (23.36%) | 1,042 (18.51%) |  | 1,280 (22.98%) | 654 (16.86%) |  |
| ***Rodent ownership at age 4 (N=9,448)*** |  |  |  |  |  |  |
| *No* | 3,331 (87.22%) | 5,016 (89.11%) | 0.005 | 4,873 (87.50%) | 3,474 (89.56%) | 0.002 |
| *Yes* | 488 (12.78%) | 613 (10.89%) |  | 696 (12.50%) | 405 (10.44%) |  |

| **Variables** | **Psychotic experiences age 13** | | | **Psychotic experiences age 18** | | |
| --- | --- | --- | --- | --- | --- | --- |
|  | **Missing**  **N (%)** | **Complete,**  **N (%)** | **P (χ²)** | **Missing**  **N (%)** | **Complete,**  **N (%)** | **P (χ²)** |
| ***Bird ownership at age 4 (N=9,448)*** |  |  |  |  |  |  |
| *No* | 3,552 (93.01%) | 5,621 (90.24%) | 0.001 | 5,195 (93.28%) | 3,683 (94.95%) | 0.001 |
| *Yes* | 267 (6.99%) | 34 (9.76%) |  | 374 (6.72%) | 196 (5.05%) |  |
| ***Turtle ownership at age 4 (N = 9,496)*** |  |  |  |  |  |  |
| *No* | 3,828 (99.66%) | 5,621(99.40%) | 0.07 | 5,576 (99.57%) | 3,873 (99.41%) | 0.27 |
| *Yes* | 13 (0.34%) | 34 (0.60%) |  | 24 (0.43%) | 23 (0.59%) |  |
| ***fish ownership at age 4 (N = 9,496)*** |  |  |  |  |  |  |
| *No* | 3,092 (80.50%) | 4,597 (81.29%) | 0.34 | 4,482 (80.04%) | 3,207 (82.32%) | 0.005 |
| *Yes* | 749 (19.50%) | 1,058 (18.71%) |  | 1,118 (19.96%) | 689 (17.68%) |  |
| ***Rabbit ownership at age 4 (N=9,448)*** |  |  |  |  |  |  |
| *No* | 3,280 (85.89%) | 4,961 (88.13%) | 0.001 | 4,792 (86.05%) | 3,449 (88.91%) | <0.0001 |
| *Yes* | 539 (14.11%) | 668 (11.87%) |  | 777 (13.95%) | 430 (11.09%) |  |
| ***Gender (N=14,661)*** |  |  |  |  |  |  |
| *Male* | 4,246 (53.35%) | 3,282 (48.97%) | <0.0001 | 5,492 (55.0%) | 2,036 (43.55%) | <0.0001 |
| *Female* | 3,713 (46.65%) | 3,420 (51.03%) |  | 4,494 (45.0%) | 2,693 (56.45%) |  |
| ***Ethnicity (N =11,993)*** |  |  |  |  |  |  |
| *White* | 5,580 (93.86%) | 5,809 (96.05%) | <0.0001 | 7,381 (94.57%) | 4,008 (95.70%) | 0.007 |
| *Non-White* | 365 (6.14%) | 239 (3.95%) |  | 424 (5.43%) | 180 (4.30%) |  |
| ***Maternal education (N=12,335)*** |  |  |  |  |  |  |
| *CSE or Vocational* | 2,399 (38.71%) | 1,310 (21.34%) | <0.0001 | 2,906 (35.96%) | 803 (18.88%) | <0.0001 |
| *Up to A levels* | 3,217 (51.91%) | 3,823 (62.28%) |  | 4,412 (54.59%) | 2,628 (61.79%) |  |
| *Degree or higher* | 581 (9.38%) | 1,005 (16.37%) |  | 764 (9.45%) | 822 (19.33%) |  |

| **Variables** | **Psychotic experiences age 13** | | | **Psychotic experiences age 18** | | |
| --- | --- | --- | --- | --- | --- | --- |
|  | **Missing**  **N (%)** | **Complete,**  **N (%)** | **P (χ²)** | **Missing**  **N (%)** | **Complete,**  **N (%)** | **P (χ²)** |
| ***Social Class (N=9,994)*** |  |  |  |  |  |  |
| *Non-Manual* | 3,503 (75.17%) | 4,501 (84.38%) | <0.0001 | 4,839 (77.29%) | 3,165 (84.78%) | <0.0001 |
| *Manual* | 1,157 (24.83%) | 833 (15.625) |  | 1,422 (22.71%) | 568 (15.22%) |  |
| ***Maternal Marital status (N= 13,387)*** |  |  |  |  |  |  |
| *Single* | 1,700 (23.69%) | 863 (13.89%) | <0.0001 | 1,977 (21.77%) | 586 (13.61%) | <0.0001 |
| *Married* | 4,970 (69.26%) | 5,046 (81.24%) |  | 6,500 (71.59%) | 3,516 (81.63%) |  |
| *Separated/Divorced/Widowed* | 506 (7.05%) | 302 (4.86%) |  | 603 (6.64%) | 205 (4.76%) |  |
| ***No. of house moves (N=7,894)*** |  |  |  |  |  |  |
| *Zero* | 313 (26.44%) | 1,247 (32.38%) | <0.0001 | 694 (9.68%) | 866 (31.97%) | <0.0001 |
| *One to three* | 400 (33.78%) | 1,456 (37.69%) |  | 817 (34.94%) | 1,039 (38.35%) |  |
| *Four to nine* | 413 (34.88%) | 1,068 (27.65%) |  | 730 (31.22%) | 751 (27.72%) |  |
| *Ten or more* | 58 (4.90%) | 92 (2.38%) |  | 97 (4.15%) | 53 (1.96%) |  |
| ***Crowding Index (N=13,095)*** |  |  |  |  |  |  |
| *0%* | 2,392 (34.19%) | 3,036 (49.79%) | <0.0001 | 3,232 (36.47%) | 2,196 (51.87%) | <0.0001 |
| *0.25%* | 2,214 (31.64%) | 1,892 (31.03%) |  | 2,828 (31.92%) | 1,278 (30.18%) |  |
| *0.75%* | 1,720 (24.58%) | 929 (15.23%) |  | 2,050 (23.14%) | 599 (14.15%) |  |
| *1%* | 671 (9.59%) | 241 (3.95%) |  | 751 (8.48%) | 161 (3.80%) |  |
| ***Stressful life events until age 4 (N=11,679)*** |  |  |  |  |  |  |
| *No* | 5,136 (93.04%) | 5,680 (92.22%) | 0.09 | 6,920 (92.94%) | 3,896 (92.04%) | 0.07 |
| *Yes* | 384 (6.96%) | 479 (7.78%) |  | 526 (7.06%) | 337 (7.96%) |  |
| ***Maternal depression in pregnancy (N=13,281)*** |  |  |  |  |  |  |
| *No* | 4,930 (82.48%) | 5,204 (87.14%) | <0.0001 | 6,500 (83.17%) | 3,634 (87.91%) | <0.0001 |
| *Yes* | 1,047 (17.52%) | 768 (12.86%) |  | 1,315 (16.83%) | 500 (12.09%) |  |
|  | **Missing**  **Mean (SD)** | **Complete,**  **Mean (SD)** | **P (F)** | **Missing**  **Mean (SD)** | **Complete,**  **Mean (SD)** | **P (F)** |
| ***Maternal Age*** | 27.44 (4.98) | 29.18 (4.52) | <0.0001 | 27.74 (4.86) | 29.42 (4.57) | <0.0001 |
| ***Paternal Age*** | 30.05 (6.97) | 30.70 (6.72) | <0.0001 | 29.98 (6.86) | 31.10 (6.76) | <0.0001 |

Table S2: Proportion of missing data by variable included in the model

| **Variable** | **Proportion missing/imputed** | |
| --- | --- | --- |
|  | **Age 13**  **%** | **Age 18**  **%** |
| **Pet* Ownership in pregnancy** | 7.73 | 8.34 |
| **Pet* Ownership at age 4** | 16.05 | 17.04 |
| **Pet** Ownership at age 10** | 13.93 | 16.19 |
| **Turtle & fish ownership at 4** | 15.66 | 16.68 |
| **Fish ownership at 10 years** | 14.38 | 16.64 |
| **Gender (Female)** | 0.04 | 0.02 |
| **Ethnicity (Non-white)** | 9.80 | 10.44 |
| **Social class (manual)** | 20.45 | 20.17 |
| **Stressful life events** |  |  |
| *18 months* | 10.87 | 12.02 |
| *30 months* | 14.56 | 15.59 |
| *42 months* | 14.18 | 15.31 |
| **Housing type (flat)** | 8.16 | 8.64 |
| **Marital Status (married)** | 7.37 | 7.89 |
| **Maternal education** | 8.46 | 9.05 |
| **Number of house moves** | 27.70 | 27.37 |
| ***Crowding Index*** | 9.05 | 9.45 |
| ***Maternal depression (yes)*** | 10.93 | 11.59 |
| ***Maternal Age*** | 7.82 | 8.49 |
| ***Paternal Age*** | 34.08 | 33.58 |
|  |  |  |

* Cat, dog, rabbit, rodent, bird

** Cat, dog, rabbit, rodent, bird, turtle

**Table S3: Univariable and multivariable Odds Ratios (OR) and 95% confidence intervals (CI) for the association between maternal cat ownership in pregnancy and between the ages of 8 months and 4 years of the child and psychotic symptoms (suspected or definite vs. none) at age 13 and 18. Sensitivity analysis using complete cases. N is specified for each exposure/outcome combination**

|  | **Psychotic experiences age 13**  **(Suspected or definite vs. none)** | | |
| --- | --- | --- | --- |
| **Exposure variable** | **Crude OR**  **(95%CI)** | **Adjusted^a^**  **OR (95%CI)** |  |
| **Cat Ownership in pregnancy (N=3,126)** |  |  |  |
| *No* | Ref | Ref |  |
| *Yes* | 1.31 (1.04 – 1.65)** | 1.34 (1.06 – 1.69)** |  |
| **Cat Ownership at age 4 (N=3,010)** |  |  |  |
| *No* | Ref | Ref |  |
| *Yes* | 1.44 (1.13 – 1.83)** | 1.47 (1.01 – 2.13)** |  |
| **Cat Ownership at age 10 (N=2,909)** |  |  |  |
| *No* | Ref | Ref |  |
| *Yes* | 1.30 (1.02 – 1.65)** | 1.15 (0.87 – 1.52) |  |
|  | **Psychotic experiences age 18**  **(Suspected or definite vs. none)** | | |
| **Exposure variable** | **Crude OR**  **(95%CI)** | **Adjusted^a^**  **OR (95%CI)** |  |
| **Cat Ownership in pregnancy (N=2,242)** |  |  |  |
| *No* | Ref | Ref |  |
| *Yes* | 1.18 (0.85 – 1.64) | 1.21 (0.86 – 1.69) |  |
| **Cat Ownership at age 4 (N= 2,161)** |  |  |  |
| *No* | Ref | Ref |  |
| *Yes* | 0.96 (0.67 – 1.37) | 0.68 (0.40 – 1.14) |  |
| **Cat Ownership at age 10 (N=2,090)** |  |  |  |
| *No* | Ref | Ref |  |
| *Yes* | 1.09 (0.77 – 1.56) | 0.90 (0.59 – 1.38) |  |

^a^= Model of cat ownership in pregnancy is adjusted for child ethnicity; maternal education, marital status, and social class; paternal age; number of house moves until age 4, type of house, crowding index. Models of cat ownership in at age four and ten years are further adjusted for cat ownership in pregnancy.

**Table S4: Univariable and multivariable Odds Ratios (OR) and 95% confidence intervals (CI) for the association between maternal cat ownership in pregnancy and between the ages of 8 months and 4 years of the child and psychotic symptoms (suspected or definite vs. none) at age 13 and 18. Sensitivity analysis using full imputed dataset (twin A). N= 15,023**

|  |  |  |
| --- | --- | --- |
|  | **Psychotic experiences age 13**  **(Suspected or definite vs. none)** | |
| **Exposure variable** | **Crude OR**  **(95%CI)** | **Adjusted^a^**  **OR (95%CI)** |
| **Cat Ownership in pregnancy** |  |  |
| *No* | Ref | Ref |
| *Yes* | 1.09 (0.92 – 1.30) | 1.13 (0.95 – 1.35) |
| **Cat Ownership at age 4** |  |  |
| *No* | Ref | Ref |
| *Yes* | 1.23 (1.03 – 1.45)** | 1.13 (0.90 – 1.42) |
| **Cat Ownership at age 10** |  |  |
| *No* | Ref | Ref |
| *Yes* | 1.20 (0.98 – 1.46) | 1.13 (0.91 – 1.41) |
|  | **Psychotic experiences age 18**  **(Suspected or definite vs. none)** | |
| **Exposure variable** | **Crude OR**  **(95%CI)** | **Adjusted^a^**  **OR (95%CI)** |
| **Cat Ownership in pregnancy** |  |  |
| *No* | Ref | Ref |
| *Yes* | 1.09 (0.87 – 1.39) | 1.15 (0.90 – 1.47) |
| **Cat Ownership at age 4** |  |  |
| *No* | Ref | Ref |
| *Yes* | 1.11 (0.87 – 1.40) | 0.92 (0.68 – 1.24) |
| **Cat Ownership at age 10** |  |  |
| *No* | Ref | Ref |
| *Yes* | 1.13 (0.87 – 1.46) | 1.05 (0.78 – 1.40) |

^a^= Model of cat ownership in pregnancy is adjusted for child ethnicity; maternal education, marital status, and social class; paternal age; number of house moves until age 4, type of house, crowding index. Models of cat ownership in at age four and ten years are further adjusted for cat ownership in pregnancy.
